# Crystal structure of botulinum neurotoxin subtype A3 cell binding domain in complex with GD1a co‐receptor ganglioside

**DOI:** 10.1002/2211-5463.12790

**Published:** 2020-01-28

**Authors:** Kyle S. Gregory, Sai Man Liu, K. Ravi Acharya

**Affiliations:** ^1^ Department of Biology and Biochemistry Claverton Down University of Bath UK; ^2^ Ipsen Bioinnovation Limited Abingdon UK

**Keywords:** botulinum neurotoxin, cell binding domain, crystallography, ganglioside binding, protein structure

## Abstract

Botulinum neurotoxins (BoNTs) are one of the most toxic proteins known to humans. Their molecular structure is comprised of three essential domains—a cell binding domain (H_C_), translocation domain and catalytic domain (light chain) . The H_C_ domain facilitates the highly specific binding of BoNTs to the neuronal membrane *via* a dual‐receptor complex involving a protein receptor and a ganglioside. Variation in activity/toxicity across subtypes of serotype A has been attributed to changes in protein and ganglioside interactions, and their implications are important in the design of novel BoNT‐based therapeutics. Here, we present the structure of BoNT/A3 cell binding domain (H_C_/A3) in complex with the ganglioside GD1a at 1.75 Å resolution. The structure revealed that six residues interact with the three outermost monosaccharides of GD1a through several key hydrogen bonding interactions. A detailed comparison of structures of H_C_/A3 with H_C_/A1 revealed subtle conformational differences at the ganglioside binding site upon carbohydrate binding.

## Graphical abstract

The majority of botulinum neurotoxin (BoNT) serotypes require recognition of both the protein and the ganglioside receptor to gain entry in to the cell. Here, we show that the cell binding domain of BoNT A3 binds three monosaccharides of GD1a receptor. Six residues of the protein interact with this receptor through several key hydrogen bonding interactions.

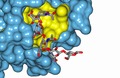

**Abbreviations**
BoNT: botulinum neurotoxin
GalNAc: N‐acetylgalactosamine
GBS: ganglioside binding site
Glu: glucose
H_C_: cell binding domain
HC: heavy chain
H_N_: translocation domain
LC: light chain
Sia: sialic acid
SNARE: soluble *N*‐ethylmaleimide‐sensitive factor attachment protein receptor

Botulinum neurotoxin (BoNT) causes the disease botulism by specifically targeting cells of the neuromuscular junction and cleaving a soluble *N*‐ethylmaleimide‐sensitive factor attachment protein receptor (SNARE) protein(s). Botulism is characterised by a descending flaccid paralysis that can be fatal without medical intervention. Considering that there are only a low number of incidences of botulism reported each year 1, there has not been a need for mass vaccination; consequently, it has been possible to use BoNT as a therapeutic for the treatment of hyperactive neuromuscular disorders. BoNTs are generally produced by *Clostridium botulinum;* however, *bont* gene clusters have recently been identified in different bacterial species 2, 3. There are currently seven distinct BoNT serotypes produced by *C. botulinum*, /A‐/G. Serotypes /A, /B, /E and /F are associated with human botulism making them potential candidates for the development of BoNT‐based therapeutics. These serotypes are further divided into subtypes (e.g., /A1‐/A8) based on minor amino acid variations that may affect toxicity 4, 5, 6. BoNTs are expressed as a single polypeptide chain (150 kDa) that is activated by post‐translational cleavage into a di‐chain consisting of a 50 kDa light chain (LC) linked to a 100 kDa heavy chain (HC) by a disulphide bond. The LC possesses zinc‐endopeptidase activity, whereas the HC comprises two domains—an N‐terminal translocation domain (H_N_) and a C‐terminal cell binding domain (H_C_).

Gangliosides constitute 10–20% of neuronal cell membranes 7 with both GD1a and GT1b present at the neuromuscular junction 8. They are amphiphilic molecules with a lipophilic ceramide tail that is inserted into the neuronal membrane, conjugated to a hydrophilic oligosaccharide moiety that is displayed extracellularly 8. GT1b and GD1a differ by only one monosaccharide, with the latter lacking the third sialic acid (Sia) (Fig. 1B). All but one BoNT serotype (BoNT/D) bind to a ganglioside receptor and a protein receptor (dual receptors) via the H_C_ domain, with the former occurring at a conserved ganglioside binding site (GBS) 9. Crystallographic studies of H_C_/A1 alone and in complex with GT1b and GD1a revealed that a majority of the interacting amino acids did not alter conformation 10, 11. Upon binding to the target cell, the BoNT is internalised into an endosome and a drop in pH triggers conformational changes in the H_N_ domain. One significant change involves a switch of buried α‐helical regions into a β‐hairpin structure that facilitates the embedding of the H_N_ into the endosomal membrane 12. The LC domain is then translocated into the cytosol of neurons at the neuromuscular junction where it catalyses the cleavage of its target SNARE protein 13. Previously we had reported the crystal structure of H_C_/A3 at 1.6 Å resolution 14; here, we report the structure of H_C_/A3 in complex with GD1a to 1.75 Å resolution and highlight the key structural changes that occur upon ganglioside binding.

**Figure 1:**
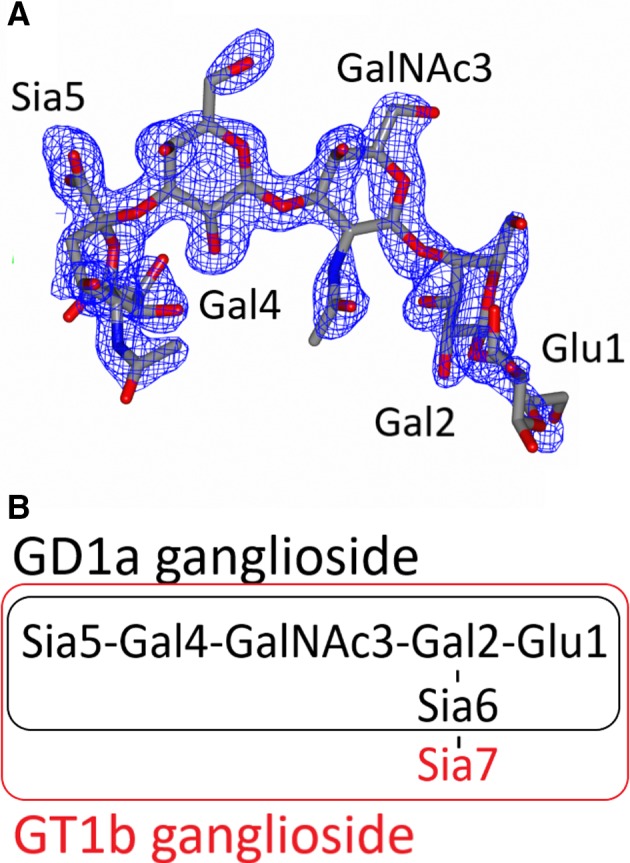
Composition of the GD1a and GT1b carbohydrate moieties. (A) Portion of Fo‐Fc electron density omit map of GD1a contoured at 3σ level. (B) GD1a and GT1b sugar moieties differ by one Sia, Sia^7^, displayed in red. Gal, galactose; Glu, glucose; GalNAc, N‐Acetylgalactosamine; Sia, sialic acid.

## Materials and methods

### Protein expression and purification

The binding domain of BoNT/A3 (residues 866–1292; ‘H_C_/A3’) was cloned into the pJ401 vector as previously described 14. The construct was transformed into BL21 *E. coli* cells and grown at 37 °C in 0.5 L TB. Cultures were induced with 1 mm IPTG upon reaching an OD_600_ of 0.6 followed by incubation at 16 °C for 16 h. Cells were lysed in 50 mm Tris pH 7.4, 0.5 m NaCl. Target protein was captured on a GE HisTrap column and further purified by size‐exclusion chromatography using a GE Superdex 200 column and 50 mm Tris pH 7.4, 150 mm NaCl.

### Protein crystallisation

Protein crystallisation was carried out using the sitting drop vapour diffusion method at 16 °C in  96‐3 well crystallisation intelli‐plates. H_C_/A3 (5 mg·mL^−1^) was added to 1.5 mm GD1a ganglioside sugar (Elicityl OligoTech) and incubated for 30 min at room temperature prior to setting up crystallisation trials with the following screens from Molecular Dimensions: PACT Premier, Morpheus I, Morpeus II, BCS, SGI and MIDAS+. Several crystal clusters formed in the BCS screen, with the best crystals observed in condition A10 (0.1 m sodium acetate, 22 % v/v PEG smear broad). These were optimised using 1 : 1, 2 : 1 and 1 : 2 protein: reservoir ratios. Crystals were mounted onto a cryo‐loop without cryo‐protection and flash‐frozen for storage in liquid nitrogen.

### X‐ray diffraction data collection and structure determination

Crystals were kept at 100 K using a liquid nitrogen jet while a total of 7200 X‐ray diffraction images were collected at 0.1° oscillations with exposures of 0.02 s using the I04 protein crystallography beamline at Diamond Light Source (Didcot, UK). Data processing was carried out using DIALS 15, and the structure was solved by molecular replacement with PHASER 16 using the structure of H_C_/A3 (PDB code: http://www.rcsb.org/pdb/search/structidSearch.do?structureId=6F0O) 14 as the search model. The model was refined with REFMAC5 17 and manually fitted in coot 18 as part of the CCP4 program suite 17. Structure validation was performed using MolProbity 19, and figures were produced using the CCP4mg molecular‐graphics software. Crystallographic data collection and refinement statistics are summarised in Table 1.

**Table 1:** X‐ray crystallographic data collection and refinement statistics. Outer shell statistics are given in brackets.

Beamline	I04 Diamond light source
Wavelength used	0.92 Å
Crystallographic statistics	
Space group	P2_1_2_1_2_1_
Unit cell dimensions	
*a*, *b*, *c* (Å)	45.23, 73.13, 140.18
α, β, γ (°)	90, 90, 90
Resolution range (Å)	140.18–1.75 (1.78–1.75)
*R*_merge_	0.217 (2.99)
*R*_pim_	0.06 (0.91)
CC_1/2_	0.996 (0.75)
<*I*/σ(*I*)>	11.2 (1.5)
Completeness (%)	99.8 (100)
No. observed reflections	1 247 184 (57 909)
No. unique reflections	47 739 (2570)
Multiplicity	26.1 (22.5)
Refinement statistics	
*R*_work_/*R*_free_	0.18/0.21
RMSD bond lengths (Å)	0.011
RMSD bond angles (°)	1.65
Ramachandran plot statistics (%)	
Favoured	96
Allowed	4
Outliers	0
Average *B*‐Factors (Å^2^)	
Protein atoms	24.98
Solvent atoms	35.12
GD1a atoms	49.07
No. Atoms	
Protein	3458
Solvent	404
GD1a	68
PDB code	http://www.rcsb.org/pdb/search/structidSearch.do?structureId=6THY

## Results and Discussion

### Crystal structure of H_C_/A3 in complex with GD1a oligosaccharide

**Figure 2:**
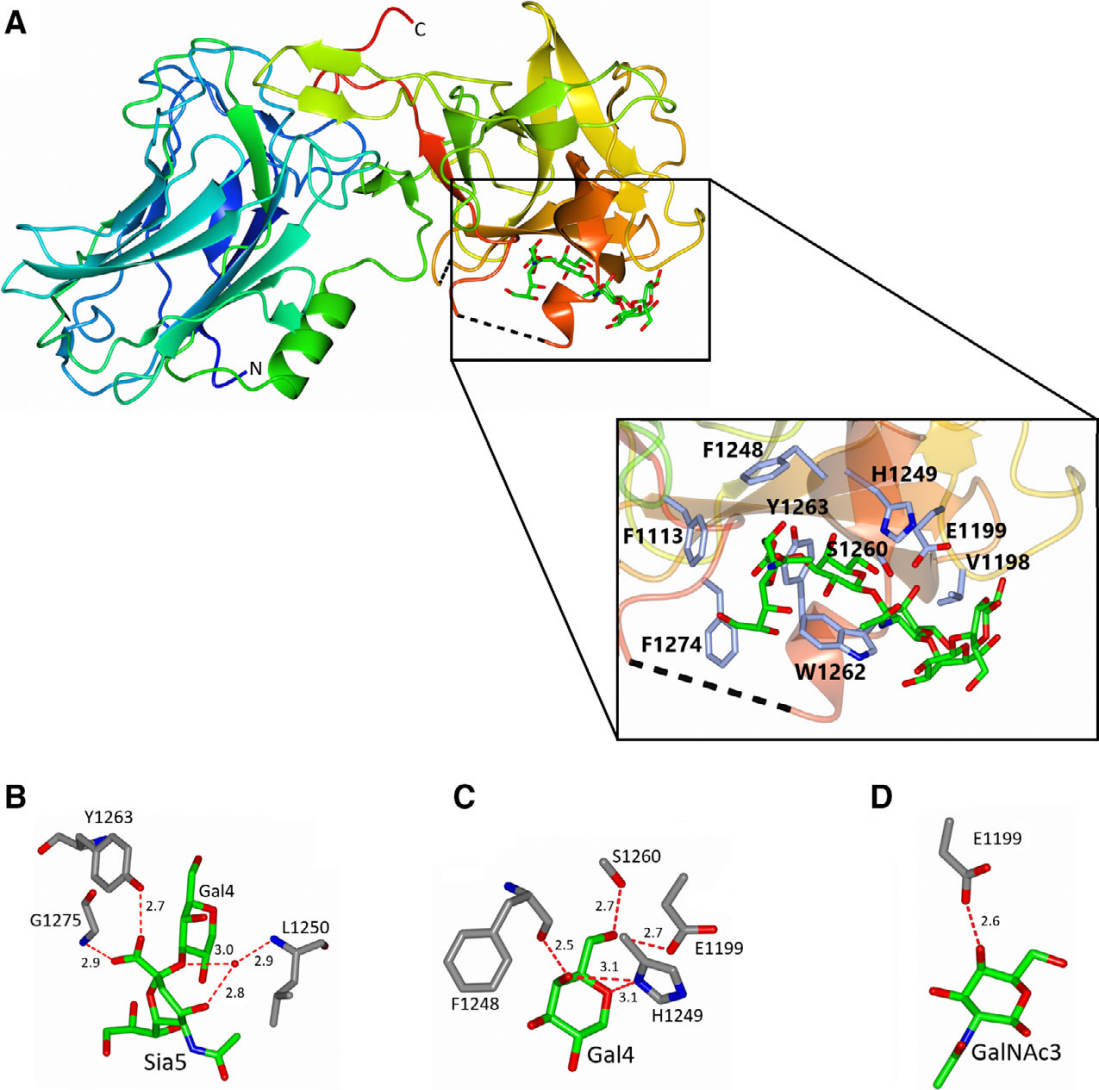
Overall structure of H_C_/A3 and interaction with GD1a. (A) Ribbon representation of H_C_/A3 in rainbow colour from N terminus (blue) to C terminus (red). The GBS is highlighted in the inset box. Unmodelled residues (residues 1222‐1228 and 1267‐1271) are represented by dotted black lines; GD1a is coloured green; H_C_/A3 side chains are coloured grey. The GBS forms hydrogen bond interactions (dotted red lines) with Sia^5^ (B), Gal^4^ (C) and GalNAc^3^ (D) A water molecule (red sphere) is involved with bridging the interaction between Leu 1250 and Sia^5^.

The crystal structure of H_C_/A3‐GD1a was solved by molecular replacement in space group P2_1_2_1_2_1_ to a resolution of 1.75 Å, with one molecule in the asymmetric unit (Table 1)**.** An initial round of refinement revealed large, positive electron density within the GBS that indicated the presence of GD1a. Monosaccharides were modelled in the observed electron density (Fig. 1A) and subsequent rounds of refinement improved the map significantly. The quality of the electron density map was very good throughout the structure, with only two small loop regions (residues 1222–1228 and 1267–1271) that were not observable. The overall fold of the protein is very similar to H_C_/A3 and other BoNT binding domain structures 10, 14, 17 where the N‐terminal half contains a 14 β‐strand ‘jelly‐roll fold’ and the C‐terminal half folds into a ‘β‐trefoil’ with a β‐hairpin that contains the conserved GBS (H..SxWY..G) (Fig. 2A). With regard to the GD1a oligosaccharide, Sia^5^‐Gal^2^ were clearly defined by the electron density and modelled with lower average B‐factors for monosaccharides interacting with the protein. Sia^5^ has a B‐factor of 45.4 Å^2^, Gal^4^ 29.2 Å^2^, GalNAC^3^ 43.5 Å^2^ and Gal^2^ 57.7 Å^2^ respectively. Glu^1^ is partially accounted for by the electron density with an average B‐factor of 73.3 Å^2^, whereas there was insufficient positive electron density to model Sia^6^.

Six residues of H_C_/A3 formed seven hydrogen bonds with GD1a (Table 2), with a conserved water molecule involved in a bridging interaction between GD1a and Leu 1250. Leu 1250 interacted with both Sia^5^ at O4 and its glycosidic bond with Gal^4^ (Fig. 2B). The hydroxyl group of Tyr 1263 formed a hydrogen bond with the carboxylic acid of Sia^5^ (2.7 Å) and the main‐chain peptide of Gly 1275 formed an additional hydrogen bond with this monosaccharide (2.9 Å) (Fig. 2B). Phe 1248, Ser 1260 and His 1249 all formed hydrogen bond interactions with Gal^4^ (2.5, 2.7, and 3.1 Å, respectively) (Fig. 2C) and glucose (Glu) 1199 formed hydrogen bonds with both Gal^4^ and N‐acetylgalactosamine (GalNAc)^3^ (2.7 and 2.5 Å, respectively) (Fig. 2C,D). Apart from these strong interactions, ring stacking interactions between Trp 1262 and Gal^4^ and GalNAc^3^ were also observed (Fig. 2A).

**Table 2:** Hydrogen bonding distances observed for ganglioside binding in H_C_/A3‐GD1a, H_C_/A1‐GT1b and H_C_/A1‐GD1a structures. Water‐mediated interactions are indicated in italics by a ‘‐H_2_O molecule (n_1_, n_2_)’ where n_1_ is the distance between the amino acid residue and the water, and n_2_ is the distance between the water and monosaccharide. ^Δ^ Indicates they are the equivalent water molecule for each structure.

Monosaccharide	H_C_/A3‐GD1a H‐bonding residue (Distance Å)	H_C_/A1‐GD1a H‐bonding residue (Distance Å)	H_C_/A1‐GT1b H‐bonding residue (Distance Å)
Sia6	Unmodelled	Trp 1266 (3.5)	Trp 1266 (3.1) *Gln 1270‐H_2_O (2.6, 2.5)* Arg 1276 (3.1)
Sia5		Tyr 1117 (2.9)	Tyr 1117 (2.8, 3.0)
	*Leu 1250‐H_2_O* ^Δ^ *(2.9, 2.8)* Tyr 1263 (2.7)	*Tyr 1267‐H_2_O (2.5, 3.5)*	
	Gly 1275 (2.9)	*Arg 1276‐H_2_O^Δ^ (2.8, 2.8)* *Gly 1279‐H_2_O^Δ^ (2.6 2.8)*	Ser 1275 (3.2) *Arg 1276‐H_2_O^Δ^ (3.1, 2.7)* *Gly 1279‐H_2_O^Δ^ (2.7, 2.7)*
Gal4	Glu 1199 (2.7)	Glu 1203 (2.8)	Glu 1203 (2.7)
	Phe 1248 (2.5)	Phe 1252 (2.7)	Phe 1252 (2.6)
	His 1249 (3.1)	His 1253 (2.7)	His 1253 (2.8)
	*Leu 1250‐H_2_O* ^Δ^ *(2.9, 3.0)*		*Gln 1254‐H_2_O (2.6, 2.5)*
	Ser 1260 (2.7)	Ser 1264 (2.8)	Ser 1264 (2.7)
GalNAc3	Glu 1199 (2.5)	Glu 1203 (2.5)	Glu 1203 (2.6) *Arg 1269‐H_2_O (2.9, 3.1)*

### Structural differences between H_C_/A3 bound and unbound to GD1a

**Figure 3:**
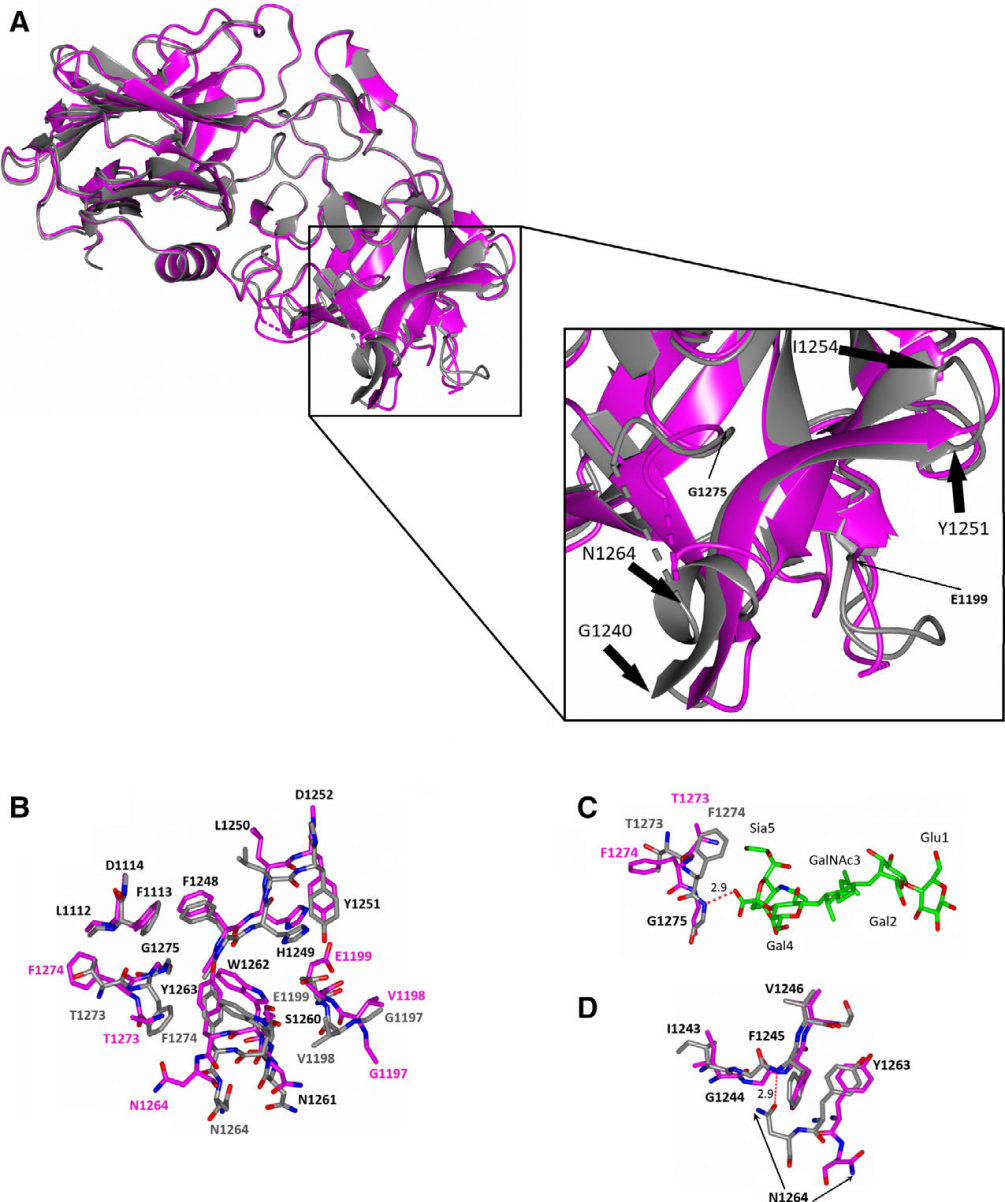
Comparison of H_C_/A3 structures bound or unbound to GD1a. (A) Superimposition (of C^α^ atoms) of H_C_/A3‐GD1a (grey) with H_C_/A3 (magenta). Unmodelled regions are represented by dotted lines, the inset box is a large‐scale representation of the GBS where the start and end of the two β‐strands of the β‐hairpin are indicated by thick black arrows, and the two residues involved in ganglioside binding that are not part of the hairpin are indicated by thin black arrows. (B) Residues within and close to the GBS of H_C_/A3‐GD1a and H_C_/A3 are shown. Those that do not deviate strikingly in position are labelled in black text. (C) The binding of GD1a (green) to H_C_/A3 involves movement of backbone to form one hydrogen bond between Gly 1275 and Sia^5^. (D) Asn 1264 flips (arrows) to form a hydrogen bond with the backbone amide of Phe 1245 upon ganglioside binding.

The structures of H_C_/A3‐GD1a (present structure) and H_C_/A3 (PDB code: http://www.rcsb.org/pdb/search/structidSearch.do?structureId=6F0O) are conformationally very similar, with an RMSD of 1.0 Å for 403 C^α^ atoms. There are, however, some noticeable differences in and around the GBS (Fig. 3A,B). Residues 1195–1196 and 1273–1277 are now clearly visible in the electron density for the H_C_/A3‐GD1a complex. The latter is located in a loop near the GBS that interacts with GD1a, which would be consistent with increased order to a flexible loop. The formation of a hydrogen bond between Gly 1275 and Sia^5^ is accompanied by flipping of positions for Phe 1274 and Thr 1273 (Fig. 3C) and loss of a water molecule.

Beyond the loop, there are additional differences in the H_C_/A3‐GD1a structure compared to the unbound H_C_/A3 structure. For example, Trp 1262 is positioned some 4 Å away from Gal^4^; Tyr 1263 has moved ~ 1.1 Å to within hydrogen bonding distance of Sia^5^, displaced a water molecule and formed a further interaction with the backbone amine of Phe 1248; and the side chain of Asn 1264 has rotated ~ 180° to form a hydrogen bond with the backbone amine of Phe 1245 (Fig. 3D). Elsewhere in the complex structure, His 1249 appears closer to the GBS and forms two hydrogen bonds with Gal^4^, and several hydrophobic residues (Phe 1113, Val 1198 Glu 1199, Tyr 1251 and Trp 1262) come together to form a shallow groove occupied by Sia^5^ → Gal^4^. This is further contributed by the C^γ^ atom of Glu 1199 that is rotated by ~ 110° about C^α^‐C^β^ bond adapting a different rotamer (Fig. 3B), and the carboxylate of this residue also forms a hydrogen bond with GalNAc^3^.

### Comparison to H_C_/A1 and H_C_/A3 structures in complex with GT1b or GD1a

Cell‐based assays have shown BoNT/A3 to have 107‐fold and 4‐fold less activity compared to BoNT/A1 in iCell neurons and HIP neurons, respectively 20. Considering that both LC and HC can effect potency separately 21, it is possible that the H_C_ domain may be partly responsible for this difference in activity between these BoNT subtypes.

**Figure 4:**
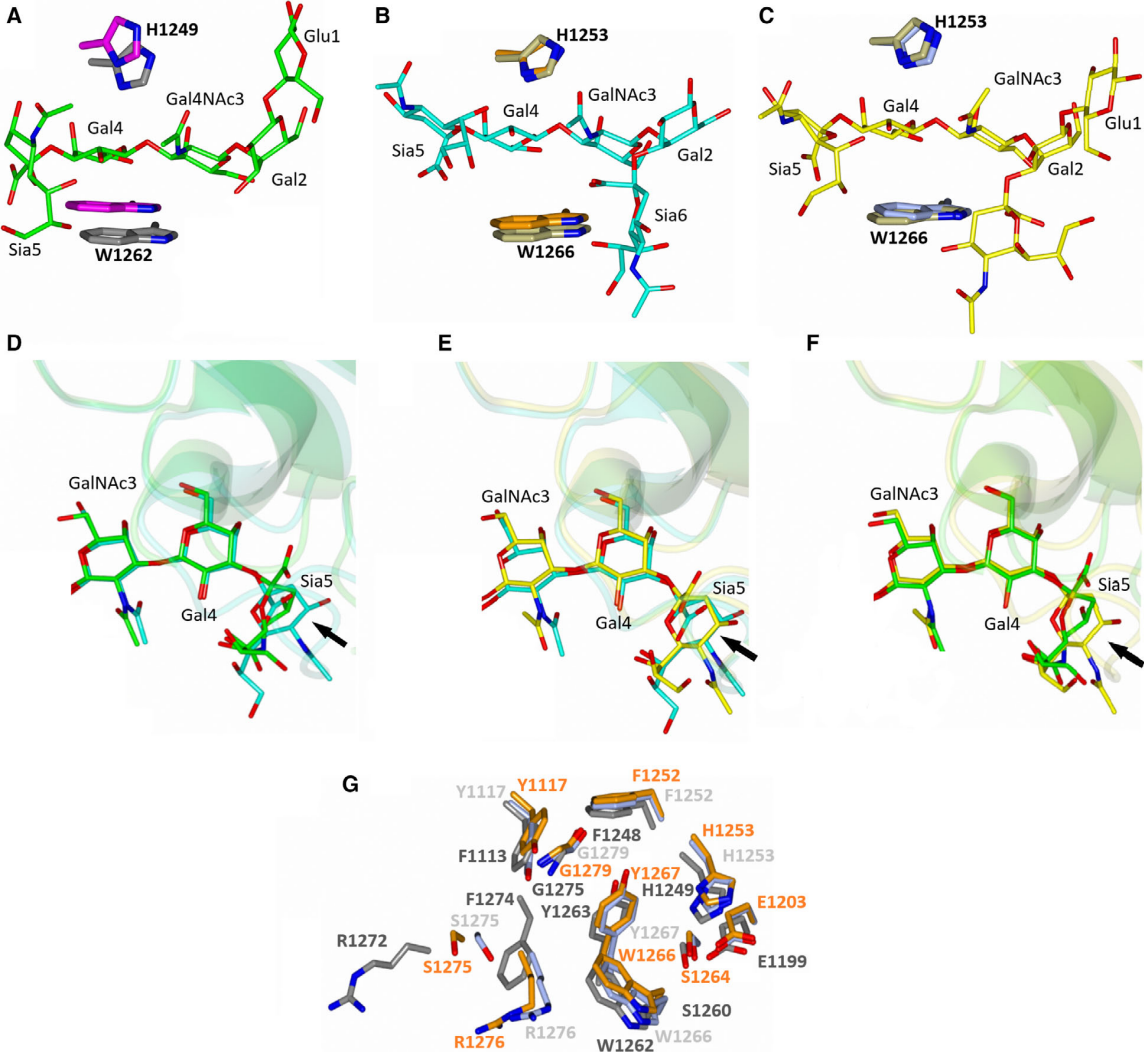
Structural comparison of the GBS for H_C_/A1 and H_C_/A3. (A) Superimposition (of C^α^ atoms) of H_C_/A3‐GD1a (grey) and H_C_/A3 (magenta) highlighting the change in position of His 1249^/A3^ and Trp 1263^/A3^ upon GD1a binding (green). (B) Superimposition (of C^α^ atoms) of H_C_/A1‐GD1a (orange) and H_C_/A1 (khaki) highlighting the change in position of His 1253^/A1^ and Trp 1266^/A1^ upon GD1a binding (cyan). (C) Superimposition (of C^α^ atoms) of H_C_/A1‐GT1b (ice blue) and H_C_/A1 (khaki) highlighting the change in position of His 1253^/A1^ and Trp 1266^/A1^ upon GT1b binding (yellow). (D) Superimposition (of C^α^ atoms) of H_C_/A3‐GD1a (green) and H_C_/A1‐GT1b (cyan) structures highlighting the difference in orientation at Sia^5^ (arrow). (E) Superimposition (of C^α^ atoms) of H_C_/A1‐GT1b (cyan) and H_C_/A1‐GD1a (yellow) structures highlighting the similarity in orientation at Sia^5^ (arrow). (F) Superimposition (of C^α^ atoms) of H_C_/A3‐GD1a (green) and H_C_/A1‐GT1b (yellow) structures highlighting the difference in orientation at Sia^5^ (arrow). (G) Superimposition (of C^α^ atoms) of residues in and around the GBS of H_C_/A1‐GD1a (colour), H_C_/A3‐GD1a (colour), and H_C_/A1‐GT1b (colour) structures highlighting the relative difference in position of selected residues.

For H_C_/A1, the structure in complex with GD1a or GT1b gives an RMSD of only 0.5 and 0.3 Å (for C^α^ atoms) compared with the uncomplexed molecule, respectively. GD1a and GT1b differ by just 1 monosaccharide (Fig. 1B) and both exhibit high affinity for the toxin 22. In both ganglioside‐bound structures, Sia^6^ is stabilised by hydrogen bonding to Trp 1266 and Arg 1276 of H_C_/A1 (Table 2), whereas for H_C_/A3, Sia^6^ could not be modelled, suggesting a lack of hydrogen bonding with the corresponding residues, Trp 1262^/A3^ and Arg 1272^/A3^. Furthermore, upon binding ganglioside, there is an accompanying shift of Trp 1262^/A3^ and Trp 1266^/A1^ in opposite directions, and together with His 1249^/A3^ and His 1253^/A1^, respectively, these residues form the opening of a groove where the ganglioside binds. For H_C_/A3, these residues have moved much farther (Fig. 4A) than those observed for the H_C_/A1 GT1b and GD1a bound structures, respectively (Fig. 4B,C). This is consistent with an induced fit mechanism for ganglioside binding where the tryptophan and histidine residues of H_C_/A3 translate ~ 7 Å.

In addition to changes in relative positions of residues after ganglioside binding (Fig. 4G), there were noticeable differences in hydrogen bonding, especially to Sia^5^. As mentioned previously, Tyr 1263^/A3^, Gly 1275^/A3^ and Leu 1250^/A3^ form hydrogen bonds with the monosaccharide, the latter of which does so through a water molecule‐bridged interaction. Although Leu 1250^/A3^ is not conserved when compared to BoNT/A1 (the corresponding residue is Gln 1254^/A1^), both ganglioside‐bound structures do display a conserved water molecule. However, the orientation of the Sia^5^ prevents a water‐mediated interaction to Gln 1254^/A1^; this difference in ring orientation is likely due to a nonconserved residue, Phe 1113^/A3^/Tyr 1117^/A1^ (Fig. 4D–F). The other two residues (Tyr 1263^/A3^ and Gly 1275^/A3^) are conserved in BoNT/A1, Tyr 1267^/A1^ and Gly 1279^/A1^, and both similarly form hydrogen bonds with the same monosaccharide in the GD1a‐bound structure, but via a water molecule. There is an additional water‐mediated and direct hydrogen bond interaction with Sia^5^ via Arg 1276^/A1^ and Tyr 1117^/A1^, respectively, which is different to that observed with H_C_/A3. For the GT1b‐bound structure, however, the Sia^5^ interactions are the same except that the water‐mediated hydrogen bond with Tyr 1267^/A1^ is replaced with a direct hydrogen bond with Ser 1275^/A1^.

### Ganglioside binding affinity may affect BoNT potency

Both H_C_/A1 and H_C_/A3 bind to three carbohydrate moieties common to GD1a and GT1b—GalNAc^3^, Gal^4^, and Sia^5^. However, considering the distinct electron density maps, greater number of interacting residues, and low average B‐factors, Gal^4^ appears to be the most tightly bound monosaccharide. Four conserved residues form hydrogen bonds to Gal^4^ with an additional water mediate interaction for Leu 1250^/A3^ and the equivalent Gln 1254^/A1^ but only for the GT1b‐bound structure (Table 2). An equivalent water molecule is present in the H_C_/A1‐GD1a structure but the position of the backbone amide hinders its interaction with the ganglioside.

Overall, H_C_/A1 forms 10 hydrogen bonds with GT1b and 7 with GD1a, while H_C_/A3 forms seven with GD1a. Similarly, H_C_/A1 has a more extensive network of water‐mediated interactions with ganglioside (four water molecules and five residues for GT1b, and two water molecules and four residues for GD1a) than H_C_/A3 (one water molecule and one residue for GD1a). This difference in combination of water‐mediated interactions and hydrogen bond interactions would be consistent with the relative ganglioside binding affinities of BoNT/A1 23, 24, 25 and suggests that BoNT/A3 binds GD1a with a lower affinity than BoNT/A1. Furthermore, the difference in degree of interaction between ganglioside and H_C_ may partly explain the reported difference in potency between BoNT/A1 and BoNT/A3 and would be consistent with the observation that BoNT/A2 has a higher affinity for gangliosides than BoNT/A1 and also enters neuronal cells more efficiently 21, 26.

## Conclusion

The high‐resolution crystal structure of H_C_/A3 in complex with the carbohydrate moiety of GD1a presented here reveals the interactions that are involved with ganglioside binding and the consequent change in conformation. A total of 6 residues form seven hydrogen bonds and one water‐mediated interaction with Sia^5^, Gal^4^ and GalNAc^3^. Although similar to H_C_/A1 binding to ganglioside, there are fewer interactions overall, mostly due to steric effects of Trp 1262^/A3^ and Arg 1272^/A3^. This would indicate a lower ganglioside binding affinity for BoNT/A3 and may be a contributing factor to its reported lower toxicity compared to BoNT/A1.

## Conflict of interest

The authors KSG and KRA from the University of Bath declare no competing financial interests. SML is an employee of Ipsen Bioinnovation Limited.

## Author contributions

KSG performed all the experiments, analysed the data and wrote the manuscript. SML analysed the data and edited the manuscript. KRA supervised the study, analysed the data and edited the manuscript. All authors reviewed the manuscript.

## Data Availability

The structure of botulinum neurotoxin subtype A3 H_C_ in complex with GD1a coreceptor ganglioside has been deposited with the RCSB‐PDB under accession code http://www.rcsb.org/pdb/search/structidSearch.do?structureId=6THY.
